# Nonmonotonic Composition Dependence of Viscosity upon Adding Single-Chain Nanoparticles to Entangled Polymers

**DOI:** 10.1021/acs.macromol.4c00206

**Published:** 2024-05-14

**Authors:** Christina Pyromali, Nikolaos Patelis, Marta Cutrano, Mounika Gosika, Emmanouil Glynos, Angel J. Moreno, Georgios Sakellariou, Jan Smrek, Dimitris Vlassopoulos

**Affiliations:** † FORTH, Institute of Electronic Structure & Laser, Heraklion 71110, Crete, Greece; ‡ Department of Materials Science and Technology, University of Crete, Heraklion 71110, Crete, Greece; § Department of Chemistry, National and Kapodistrian University of Athens, Panepistimiopolis, Zografou, 15771 Athens, Greece; ∥ Dipartimento di Ingegneria Chimica e Materiali, Università Degli Studi di Cagliari, Piazza D’Armi, I-09123 Cagliari, Italy; ⊥ Centro de Fisica de Materiales (CSIC-UPV/EHU) and Materials Physics Center MPC, Paseo Manuel de Lardizabal 5, E-20018 San Sebastian, Spain; # Department of Physics, School of Advanced Sciences, Vellore Institute of Technology, Vellore 632014 Tamil Nadu, India; ¶ Donostia International Physics Center, Paseo Manuel de Lardizabal 4, E-20018 San Sebastian, Spain; ∇ Faculty of Physics, University of Vienna, 1090 Vienna, Austria

## Abstract

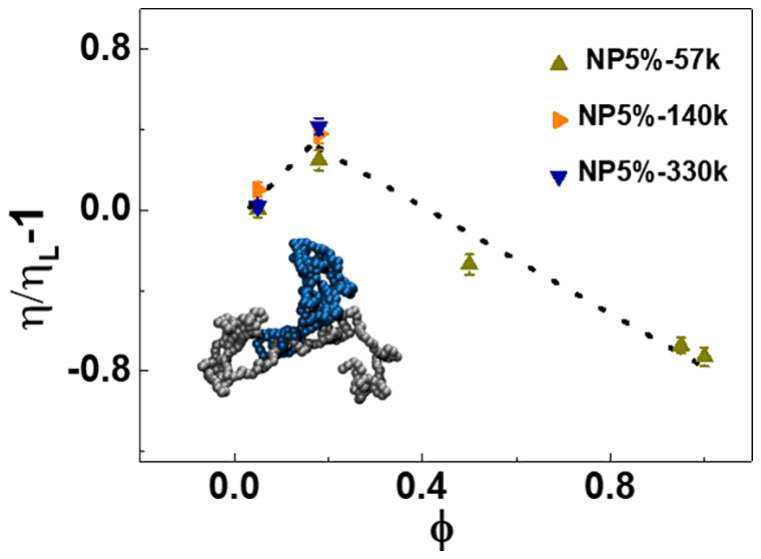

Well-characterized single-chain nanoparticles (SCNPs), synthesized from a linear polystyrene precursor through an intramolecular [4 + 4] thermal cycloaddition cross-linking reaction in dilute conditions, were added to entangled polystyrene melts at different concentrations. Starting from the pure linear melt, which is much more viscous than the melt of SCNPs, the zero-shear viscosity increased upon the addition of nanoparticles and reached a maximum before eventually dropping to the value of the SCNP melt. Molecular simulations reveal the origin of this unexpected behavior, which is the interplay of the very different compositional dependences of the dynamics of the two components. The SCNPs become much slower than the linear chains as their concentration decreases because they are threaded by the linear chains, reaching a maximum viscosity which is higher than that of the linear chains at a fraction of about 20%. This behavior is akin to that of single-loop ring polymers when added to linear matrices. This finding provides insights into the design and use of SCNPs as effective entropic viscosity modifiers of polymers and contributes to the discussion of the physics of loopy structures.

## 1 Introduction

The synthesis of well-characterized single-chain nanoparticles (SCNPs), or self-associating chains, has received a great deal of attention in the last two decades by attempting to mimic the functionality and precision of folded biomolecules such as intrinsically disordered proteins (IDPs) and enzymes.^1,2^ Single polymer chain folding via intramolecular cross-linking results in SCNPs with tunable structure-motion-performance interplay. Sparse SCNP morphologies resemble IDPs functionality, while single-domain globular SCNPs mimic high enzymatic-like catalytic efficiency. The soft nature of SCNPs provides the possibility of external confinement in the bulk state or in nanocomposites.^3−6^ Intramolecular self-confinement restricts the degrees of freedom and compactifies a polymer coil similarly to ring polymers, resulting in distinctly different properties from those of the linear precursor.^6^ Precisely synthesized soft nano-objects exhibit a number of superior biosensing, biocatalytic, and rheological properties related to loop formation. In analogy to ring polymers, SCNPs exhibit a multiloop topology, which reflects the interplay of the intramolecular cross-linking density and the length of the linear parent polymer.^5,7−9^

Experiments with SCNP solutions revealed compact morphologies and a reduced translational diffusion coefficient with respect to their linear precursors.^3,10,11^ Molecular dynamics (MD) simulations showed that SCNPs with sparse morphologies at high dilution (intrinsically sparse SCNPs with scaling exponent ν ≈ 0.5) change to crumpled globular objects (ν ≈ 1/3) as the concentration of SCNPs increases beyond overlap.^11^ Characterizing pure SCNPs in the melt state can be challenging because even traces of reactive groups might lead to irreversible interparticle cross-linking with time.^5,6^ Despite their soft nature, globular SCNPs melts (considered to be the upper limit of self-confinement) exhibit slow gel-like relaxation similar to jammed colloidal systems.^12,13^ On the other hand, recent experiments suggest that pure SCNPs relax stress faster than their linear counterparts due to the reduced intermolecular constrains,^7,14^ which in the framework of tube models may be interpreted as an effective reduction of the number of entanglements. These observations are in agreement with MD simulations of intramolecularly cross-linked SCNPs.^7,14−17^

SCNPs have been utilized as nanofillers in linear chains to yield all-polymer nanocomposites. Several groups have reported the viscosity reduction of entangled polymer chains in the presence of nanoparticles.^12,18−21^ Recent experimental and simulation studies have linked the decrease in viscosity to the reduction of friction in blobs with similar size to SCNPs. Actually, the viscosity decreased dramatically in nanocomposites with longer matrix chain lengths.^22,23^ Despite these developments, an outstanding challenge emerging from the above remains fundamentally understanding the dynamics of SCNPS blended with linear matrices. Here, we focus on the loopy structure of SCNPs and their interplay with linear chains, with the aim to establish a link with the behavior of ring–linear polymer blends^8^ and exploit the consequences of loop threading. To this end, we use well-characterized SCNPs and add them to entangled linear polymer melts in order to address their linear viscoelastic response.

## 2 Materials and Methods

### 2.1 SCNPs and SCNP-Linear Polymer Blends

**Figure 1:**
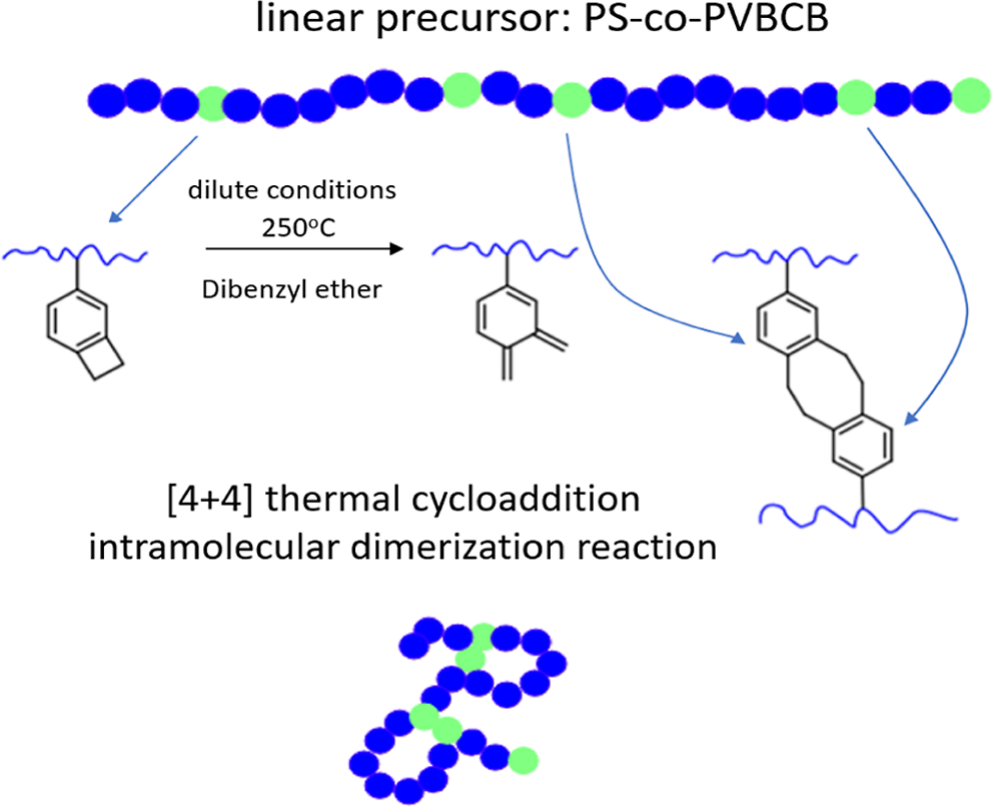
Schematic of random copolymer PS-*co*-PVBCB folding into SCNP.

The linear random polystryrene-*co*-poly4-vinylbenzocyclobutene copolymer (PS-*co*-PVBCB) was prepared by nitroxide mediated radical polymerization together with benzocyclobutene (BCB) chemistry.^24^ The SCNPs were synthesized from the linear precursor through an intramolecular [4 + 4] thermal cycloaddition dimerization reaction under dilute conditions at 250 °C. Further details regarding the synthetic approach and the molecular characteristics of the SCNPs are provided in ref (25) (see also Figure 1) and in the Supporting Information, where size exclusion chromatography (SEC) eluograms are also presented (Figures S1–S3). The conversion of the intramolecular cross-links was confirmed by the ^1^H NMR spectra of Figure S4. Figure S5 schematically illustrates the SCNPs. Molecular control over the structure, morphology, and folding behavior can be achieved by changing the cross-linker fraction (CrF) and/or the length of the precursor copolymer. The morphology of the resulting nanoparticles depends on both the number-average molar mass *M*_n_ and the CrF of the copolymer precursor. Here, we fix the CrF to a relatively low value of 5% (yielding a rather sparse nanoparticle morphology with loops clearly exceeding the Kuhn molar mass) and change the *M*_n_ (57, 140, and 330 kg/mol), but the cross-link density distribution cannot be controlled. The molecular characteristics of the SCNPs are given in Table 1.

**Table 1:** Molecular Characteristics of SCNPs Used

SCNP code	% BCB^a^ (mol)	*M*_n,prec_^b^ (kg/mol)	PD^c^	*R*_h_^d^ (nm)	*n*_loops_	*M*_w,loop_ (kg/mol)
5%-57k	4.8	57	1.17	5.3	14	4.17
5%-140k	4.9	140	1.25	7.9	34	4.17
5%-330k	4.8	330	1.37	9	79	4.17

a The cross-linker fraction is determined by ^1^H NMR in CDCl_3_.

b The number-average molar mass of the linear precursors PS-*co*-PVBCB is determined by size exclusion chromatography (SEC) in CHCl_3_ at 25 °C (calibration with PS standards).

c The respective polydispersity of the linear precursors PS-*co*-PVBCB is determined by SEC.

d The hydrodynamic radius is determined by dynamic light scattering in the good solvent CHCl3 at 25 °C. Note that the shape factors *R*_g_/*R*_h_ of similar SCNP polystyrenes have been reported by Tuteja et al. in the good solvent THF.^12^ It was found that for lightly cross-linked nanoparticles (2.5% cross-linker), *R*_g_/*R*_h_ is close to 1.2 (Gaussian coil regime), while for tightly cross-linked nanoparticles (20% cross-linker), *R*_g_/*R*_h_ is below 1 (approaching the hard sphere regime).

Blends of different SCNPs with linear PS chains at various weight fractions from 4.7 to 95 wt % (see Table S1 and Figure S6) were prepared by dissolving the desired amount of SCNPs and linear PS in THF (3.75% w/v) at room temperature. The solutions were stirred gently until becoming macroscopically homogeneous and were then initially dried in ambient conditions (this was monitored by measuring the weight of the sample) and then at 40 °C under vacuum. Subsequently, the samples were annealed under dynamic vacuum at 110 °C overnight to facilitate solvent removal and were finally shaped into discotic specimens of 0.7 to 1 mm thickness using a stainless-steel vacuum mold.

### 2.2 Rheology

Linear viscoelastic (LVE) measurements were performed on an ARES strain-controlled rheometer (TA Instruments, USA) equipped with a force rebalance transducer (2KFRTN1) and nitrogen flow convection oven with temperature control of ±0.1 °C. Stainless-steel parallel plates with a 4 or 8 mm diameter were used. The measurement protocol is detailed in the Supporting Information (see also Figures S7–S10). Equilibration of the sample was ensured by performing a dynamic time sweep (DTS) measurement for at least 30 min at each temperature, while the gap was adjusted according to the thermal expansion coefficient of the tools. The LVE regime was determined based on dynamic strain sweep (DSS) experiments at each temperature. Subsequently, small-amplitude oscillatory shear measurements at a fixed linear strain amplitude and a frequency range of 10^2^–10^–1^ rad/s (dynamic frequency sweeps, DFS) were performed in the temperature range 105–170 °C. The data are presented in the form of storage and loss moduli, *G*′ and *G*″, respectively, as a function of oscillatory frequency. In order to ensure the thermal stability of the samples, we deployed sensitive phase angle shift (δ) representations and a suitable heating protocol, where the lower temperatures were measured first. For example, a loosely cross-linked (CrF = 5%) NP with *M*_n_ = 330k of the parent copolymer exhibits a discrepancy in the vGP representation that is related to interparticle coupling events at temperatures over 170 °C, most likely due to the presence of reactive groups after synthesis (Figure S7). LVE master curves (shown in the paper and in Figures S8 and S9 at the same distance from *T*_g_) were obtained by applying the time–temperature superposition (TTS) principle. The TTS validity confirmed the thermorheological simplicity of the samples. This is further confirmed by the sensitive van Gurp-Palmer representation of the LVE data in terms of loss angle versus complex modulus (Figure S7, for SCNP-linear polymer blends). Master curves were constructed at a reference temperature of *T*_ref_ = 150 °C and then shifted to a constant distance from the corresponding *T*_g_, at *T* = *T*_g_ + 24 °C, ensuring that the high-frequency data match in order to compare the different LVE data at iso-frictional conditions. Figure S10 depicts the horizontal (*a*_T_) and vertical (*b*_T_) shift factors as a function of the temperature. The former were fitted with the WLF equation,^26^, to yield the coefficients *C*_1_ = 7 and *C*_2_ = 67.13 °C at *T*_ref_ = *T*_g_ + 24 °C. The vertical shifting accounts for the density change with temperature for PS.^27^

### 2.3 Simulations

Molecular dynamics (MD) simulations were performed using the Kremer-Grest bead–spring model.^28^ The precursors were linear chains of *N* = 200 beads. Fifty beads (randomly selected) were reactive (with the condition of nonconsecutive reactive beads to prevent trivial cross-links). The nonbonded bead–bead interactions were given by purely repulsive Lennard-Jones (LJ) potentials, mimicking purely excluded volume interactions. After the reactive sites were permanently cross-linked to produce the SCNPs, simulations of linear/SCNP blends with different SCNP fractions were performed at the same total (melt) density.

The LJ diameter σ (bead size) will be used as the size unit and qualitatively corresponds to a Kuhn length (∼1 nm). Chain connectivity and bond uncrossability were implemented by FENE potential.^28^ We included a moderate bending potential to implement some bending stiffness, which increases the number of entanglements, *Z*, per chain. Primitive path analysis of the melts of these linear chains yields an approximate value of *Z* = 8.^29^ We created the SCNPs through MD simulations of the isolated chains (so cross-linking was purely intramolecular by construction, and obviously, all SCNPs had 200 beads). The reactive sites were monovalent; i.e., when two reactive sites found each other within the (short) capture radius, they formed a permanent FENE bond and were not allowed to form other bonds with other sites. Further, we created a set of SCNPs constructed in the former way. As expected, they were topologically polydisperse, and their morphologies were mostly sparse. Then, we selected some of them and put them in a large simulation box, imposing intermolecular minimum distances that strictly prevented the catenation of loops of different SCNPs. Further, we inserted the linear chains of *N* = 200 beads through chain growth and rejection of bead overlaps. The system was very slowly compressed and equilibrated at the desired density (total number of beads/box volume ρ = 0.85, which qualitatively corresponds to melt conditions^28^). All the simulated systems had the same density, interactions, and number of beads per molecule (SCNP or linear). Therefore, dynamic differences were strictly due to topological effects, which were tuned by changing the fraction of SCNPs (ϕ) in the SCNP/linear blend. The time unit of the scattering functions qualitatively corresponds to 1 ps.^28^ We simulated the cases ϕ = 0 (pure linear), ϕ = 0.10, 0.25, 0.35, 0.50, 0.65, 0.80, and ϕ = 1 (pure SCNPs). Further details of the model and simulation method can be found in refs (11,29). It should be noted that ref (29) was focused on blends of linear chains with intrinsically globular SCNPs (compact spherical nanogels even at high dilution), where a slightly negative χ-parameter was imposed in the cross-interaction to prevent demixing of SCNPs and linear chains. In our blends, the SCNPs are intrinsically sparse, and all (self- and cross-) interactions were identical (χ = 0), which was enough to have good mixing.^11^

## 3 Results and Discussion

### 3.1 Linear Viscoelasticity and Dynamics of SCNP-Linear Polymer Blends

**Figure 2:**
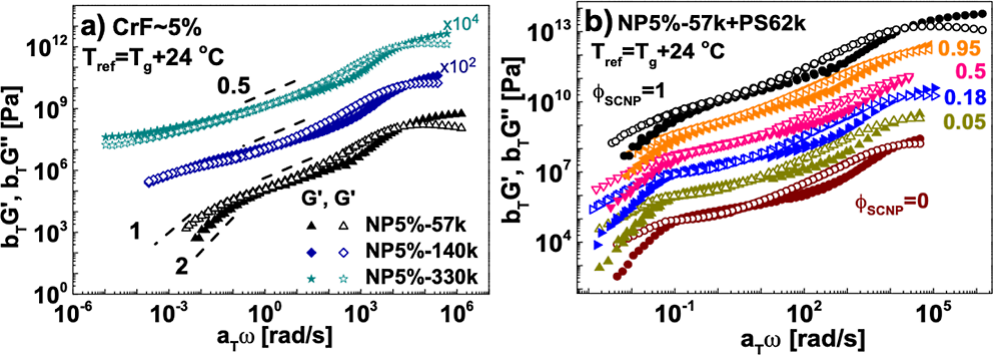
(a) LVE master curves of *G*′ (closed) and *G*″ (open) against angular frequency for SCNP melts with nearly constant CrF and different parent PS-*co*-PVBCB lengths (57, 140, and 330k). The data are shifted to match the high-frequency regime at the iso-*T*_g_ condition. The curves are shifted vertically, as indicated on the plot, for clarity. (b) Dynamic master curves of *G*′ (closed) and *G″* (open) against frequency for pure polymers and blends at different mass fractions (5, 18, 50, and 95%) of SCNP (5%-57k) at iso-*T*_g_ condition (the data are shifted vertically with respect to the linear matrix ϕ_SCNP_ = 0, up by one decade for each mass fraction, to appreciate the extent of the power-low relaxation).

The intramolecular cross-linking of the 5% mol % CrF linear random precursor PS-*co*-PVBCB with *M*_n_ = 57 kg/mol into a sparse SCNP causes the acceleration of the dynamics in comparison to linear polystyrene with a similar degree of polymerization (LPS62k). Intramolecular loop formation in SCNPs reduces the intermolecular topological constraints and, thus, the effective entanglement contacts in the melt state, similar to other cyclic polymers.^5,7,8^ In particular, it was recently shown that SCNPs exhibit suppressed broadness of relaxation and magnitude of plateau modulus in comparison to the parent polymer.^14^ This effect is illustrated for our systems in Figure S9 in Supporting Information, which compares SCNP melts at a fixed CrF of 5% and different *M*_n_ values. The constant fraction of the cross-linker is translated into the same average length of loops, and the longer parent PS-*co*-PBCB into a larger number of loops. The LVE moduli are depicted in Figure 2a. The unchanged LVE moduli from high to intermediate frequency regimes (see Figures S7–S9) provide evidence to support the argument that the length of the loops remains unchanged when CrF is kept constant. In this context, the Rouse-like slope of 0.5 at intermediate frequencies reflects internal loop relaxation (loops are clearly unentangled; see Table 1). Stress relaxation proceeds in a hierarchical manner in the sense that loops and trapped PS subchains exhibit fast local relaxation, which is then followed by the eventual center of mass motion of the SCNPs. Depending on the degree of interlocking, the SCPNs may exhibit a low-frequency soft colloidal plateau modulus,^13,14^ typical of jammed systems, before terminal relaxation. As discussed above, the 5%-57k nanoparticle forms a smaller number of loops that rapidly relax, and the contribution of the free chain end fluctuations relaxation is also important due to the higher free end density. The intermediate length in the parent polymer slows the dynamics down because of the increase in the number of loops, which promotes the creation of multiloop domains. The nanoparticle 5%-140k rubbery-like response is interpreted as an ultrasoft soft colloidal response. In the extreme case of a precursor length of 330k of loosely cross-linked SCNP, the enhanced interlocking is reflected in the low-frequency solid-like response (jamming). The interlocking via loops in SCNPs promote local heterogeneities, as confirmed by dielectric spectroscopy data for SCNPs with 140k and 330k, which indicate heterogeneous segmental dynamics.^25^

The above loosely cross-linked SCNPs were blended with linear PS of different molar masses and at different concentrations, with the aim to investigate the influence of the intramolecular structure on the rheological properties of all-polymer PS nanocomposites. The dynamic moduli and the corresponding complex viscosities as a function of frequency for blends with different mass fractions of the loose SCNP 5% −57k nanoparticles are shown in Figure 2b.

**Figure 3:**
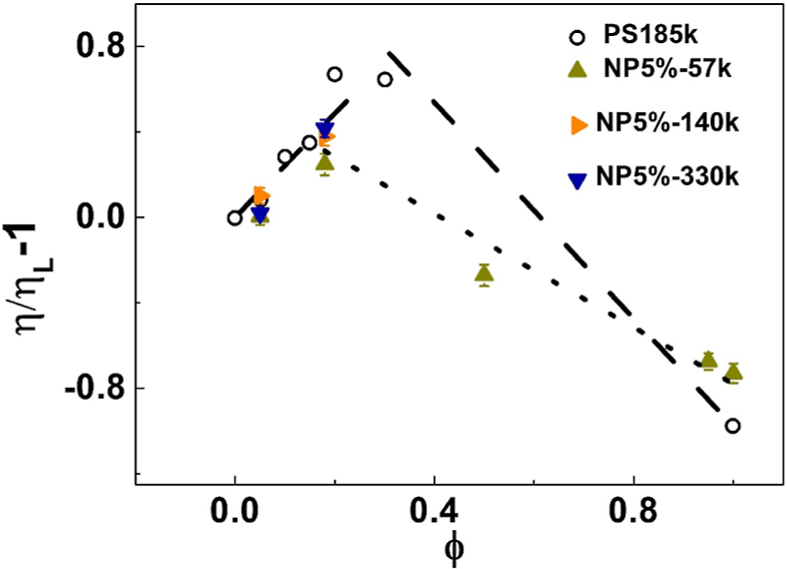
Relative viscosity increment (with respect to the pure linear chains) of loose SCNP-linear blends as a function of the SCNP mass fraction ϕ of SCNPs melts at a fixed CrF of 5%r (triangles, see legend). On the same plot, respective data (circles) for symmetric ring-linear blends (with a molar mass 185,000 g mol^–1^) as a function of ring mass fraction (taken from ref (8)) are included. The data for both types of blends are presented at iso-frictional conditions (*T*–*T*_g_ = 46.5 °C). The lines are drawn to guide the eye (short dashes for SCNP-linear blends and long dashes for ring-linear blends).

The zero-shear viscosity of the SCNP 5%-57k melt is lower than the viscosity of the linear matrix, while for the corresponding nanocomposites, the zero-shear viscosity exhibits a nonmonotonic behavior with the SCNP weight fraction (Figure 3). Entropically driven loop threading has recently been reported in the combined experimental and modeling study of symmetric ring-linear blends.^8^ It was found that the addition of a low fraction of (faster) rings increased the viscosity of the (slower) linear matrix due to threading. Indeed, the rings can relax solely by a constraint release process that is activated by linear chain reptation. The relative viscosity increment (with respect to linear chains) of symmetric ring linear blends and loose SCNP-linear blends with the same loop length (CrF = 5%) are shown in Figure 3. Compared to ring-linear blends, the present SCNP-linear blends can be considered asymmetric in the sense that the loop size of the SCNPs is smaller than the linear chains. But the similarity of the two blends, exhibiting nonmonotonic composition dependence on their zero-shear viscosity, is unambiguous.

**Figure 4:**
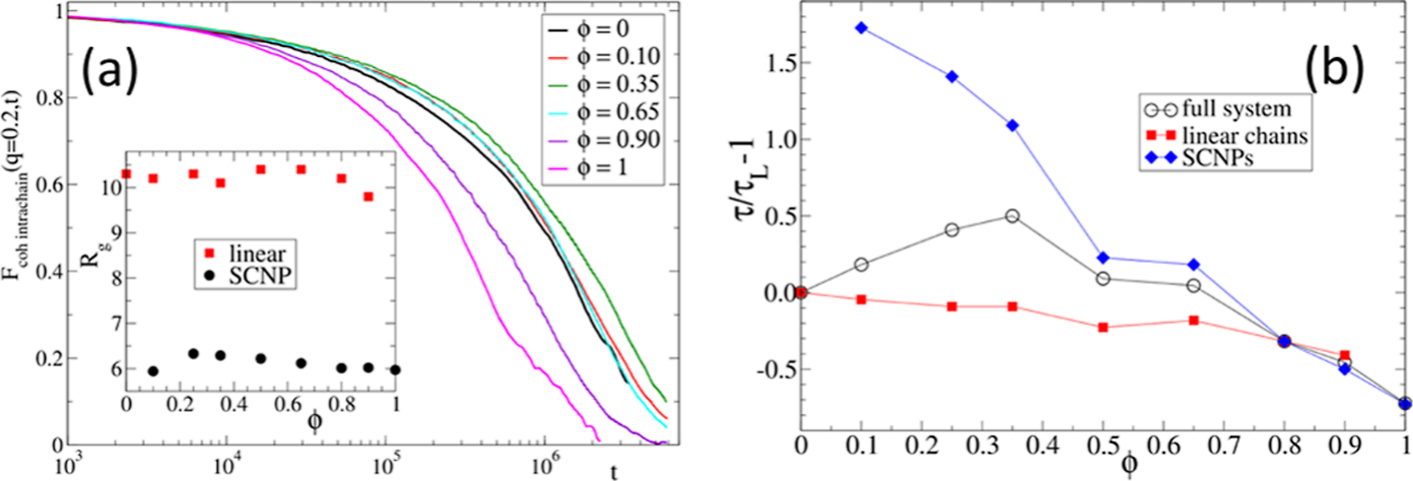
(a) Total intramolecular coherent scattering function for a blend of SCNPs added to linear chains at different compositions from ϕ = 0 (pure linear) to ϕ = 1 (pure SCNPs). Inset: calculated *R*_g_ for SCNP and linear chain as a function of the blend’s composition. (b) Variation of the relaxation times (τ) of linear, SCNP, and blend (total), extracted from the respective partial and total intramolecular coherent functions (the times are normalized with the relaxation time of the pure linear melt, τ_L_ = 2.2 × 10^6^, to represent a relative increment analogous to Figure 3). An illustration of threading is included in the Supporting Information.

We now attempt to rationalize the experimental data with the help of MD simulations. The total intramolecular coherent scattering function for several SCNP-fractions, normalized to 1 at time *t* = 0, is shown in Figure 4a. It is computed as the standard coherent scattering function by considering only pairs of beads belonging to the same molecule. Data are shown for the wavevector *q* = 0.2, which corresponds to a distance of 2π/*q* ≈ 30, i.e., approximately 3 and 5 times the *R*_g_ of the linear chains and the SCNPs, respectively. Therefore, its relaxation time probes diffusion time scales and should be proportional to the viscosity. A nonmonotonic behavior with the blend composition is observed, similar to the viscosity in the experiments. The normalized partial intramolecular coherent scattering functions (averaged over all the linear chains or all of the SCNPs) were computed and are shown in Figure S12 (the function for the total system is just the weighted average of the two partial ones). The inset of Figure 4a depicts the mean radius of gyration (*R*_g_) of each component (linear or SCNP) of the blend, which is considered to be virtually unaffected by the composition of the blend. The observed very weak dependence of both *R*_g_ values on SCNP concentration could even be a statistical artifact since the SCNPs are topologically polydisperse and different in each blend due to the random procedure of cross-linking. The extracted relaxation times τ of the total and partial functions, which have been defined as the time for which they decay to 0.25 (roughly corresponding to the time extracted by a single exponential fit), are depicted as a function of the SCNP concentration in Figure 4b. Similarly to Figure 3, the data are represented in terms of variation with respect to the relaxation time of the pure linear system. Remarkably, the normalized time of the total function follows the nonmonotonic dependence on the SCNP concentration found for the experimental viscosity, even semiquantitatively, with a maximum at ϕ ≈ 0.35. Of course, the relaxation time of the total function originates from the interplay of the partial times of the linear chains and SCNPs, which we discuss next. In consistency with results reported in the literature,^14,29^ the pure melt of SCNPs relaxes much faster than its pure linear counterpart at the same density and molecular weight. This is attributed to the combination of high penetrability and the small size of the intrinsically sparse SCNPs. Because of their high penetrability, they do not experience caging in their dynamics, as opposed to the weakly penetrable intrinsically globular SCNPs. Their small size suggests fewer intermolecular contacts than the linear chains. Concerning the time extracted from Figure 4a, it should be noted that some apparent long-time features are observed in some scattering functions. They might be just a statistical artifact related to the intrinsic polydispersity of the SCNPs, and to address this point, more simulations would be needed in the future. However, irrespective of how the relaxation time is defined, the curves in Figure S12b exhibit an unambiguous monotonic behavior with ϕ, so the qualitative trends in the times seen in Figure 4b are not affected by the specific choice.

As expected, adding intrinsically faster SCNPs leads to a progressive acceleration of the relaxation of the linear chains. Concomitantly, adding intrinsically slower linear chains leads to a progressive slowing of the SCNPs. The nonmonotonic behavior of the total relaxation time results from the interplay of the different dependences of the partial times on the blend composition, which is much stronger for the SCNPs’ times (see Figure 4b). What is surprising is that, for ϕ < 0.8, the SCNPs become slower than the linear chains, even by a factor of 3 at ϕ = 0.1. This result cannot be explained by topological effects associated with entanglements. Instead, it is due to (and represents a signature of) the threading of SCNPs by linear chains. In ref (29), simulations for blends of linear chains and intrinsically globular SCNPs were analyzed in the framework of the tube model. Unlike in our case, where the SCNPs are sparse objects at high dilution and only become crumpled globules in crowded conditions, in ref (29) the SCNPs were spherical nanogel-like objects by design and therefore much less deformable and penetrable by neighboring chains. These soft nanofillers led to a reduction in the effective entanglement length of the linear chains. Threading of the SCNPs by the linear chains was suggested to occur, but it was not quantified. Moreover, such SCNPs could not diffuse in the simulation time scale, effects on the viscosity could not be investigated, and no direct comparison with experiments was given. We shed light on the former questions by analyzing dynamic data and threading events in our simulations.

### 3.2 Threading Analysis

**Figure 5:**
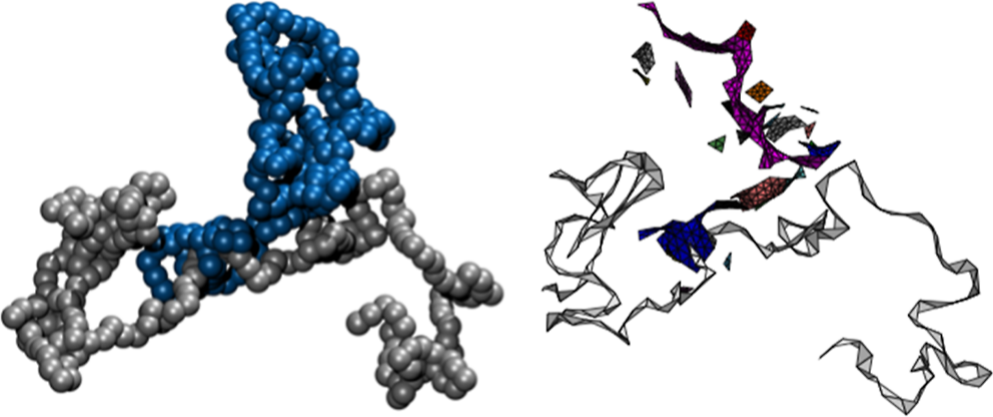
A linear chain threading a SCNP. Left: snapshot from the MD simulation SCNP in blue, L in gray. Right: the same snapshot was represented with minimal surfaces: the colored triangulated surfaces represent the 25 cycle-basis members of the SCNP. Note that they are not all connected to each other because the interconnecting linear segments are not shown. The blue surface is pierced by a linear chain. For visualization purposes only, the gray-colored linear chain is made “thicker” by spanning a triangular facet on every three consecutive beads.

To prove and quantify the existence of the threadings, we analyzed about 150 equilibrium simulation snapshots using a method of minimal surfaces that has been developed in the context of ring polymers.^30,31^ In essence, we compute a triangulated minimal surface using the Surface Evolver software,^32^ spanned on a contour of each ring, and calculate geometrically when a segment of a linear polymer pierces any triangle of the surface. Such a situation we call threading (see Figures 5 and S7).

The advantage of the use of the minimal surface in comparison to other possible spanning surfaces is that the minimal surface is well-defined and, importantly, lies completely “within” the volume spanned by the ring, thereby giving threading the intuitive geometrical interpretation of piercing through the interior of the ring.^30−32^Figure 5 illustrates a representative threading event (of SCNP by a linear chain) from our simulations. The peculiarity of SCNPs in comparison to plain rings is the fact that each SCNP contains a number of distinct loops due to the cross-links.

To identify the elementary cycles for each nanoparticle, we constructed a cycle basis of its corresponding graph using the algorithm implemented in the NetworkX package version 3.1 in the Python language.^33^ The graph’s nodes are the monomers, and the edges of the graph are defined by the linear connectivity and the cross-links. The cycle basis is a minimal set of cycles that can form any cycle in the graph by summation of the cycles from the basis set.^33^ The summation is meant as an “exclusive or” operation on the edges. Because the reactive sites are monovalent and nonconsecutive, there are always 25 cycles in the basis (for a general graph, it is #edges-#nodes-#connected_components); therefore, for each nanoparticle, we have 25 “rings” that can be threaded by the linear chains (here, #edges refers to the counted number of edges, etc.).

For each member of the basis cycle (ring), the initial surface of disk topology is constructed as a union of triangles where each has two vertices fixed on two consequent ring monomers, and the third is in the center of mass of the ring. The initial surface of N triangles is then once refined (each triangle is divided into 4 finer triangles by introducing extra vertices at the edge centers). The surface of 4N triangles is then minimized by moving the free vertices (those not belonging to the boundary) under the effect of an overdamped surface tension force using the Surface Evolver software,^32^ as described in detail in ref (31). The potential convergence complications reported in ref (31) for longer rings do not arise in the present study as the rings are relatively short. See an example threading event in Figure 5.

Note that the threadings of distinct rings from the cycle basis can be dependent in the sense that if, for example, ring B is threaded, then ring A is also threaded. Such a situation can arise when a significant fraction of ring B forms a subset of ring A, and the rings are relatively flat, so their minimal surfaces do not differ significantly (see Figure S11 for a sketched example). The threading statistics we extract are independent of this feature.

**Figure 6:**
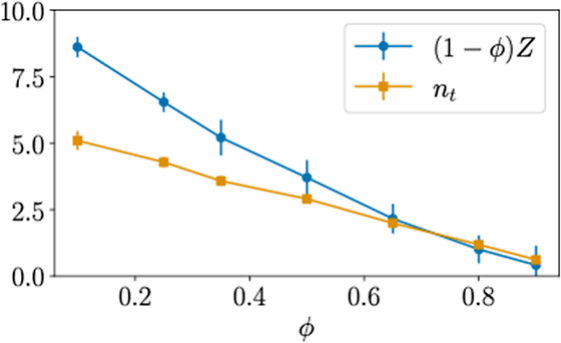
Number of linear chains threading each SCNP on average (*n*_t_) as a function of SCNP composition ϕ. For comparison, the number of linear–linear entanglements (1-ϕ)*Z* is also shown. Symbols represent the mean over snapshots and the error bars the error of the mean.

We define a SCNP to be threaded by a linear chain if the chain pierces at least one of the corresponding cycles (which form the SCNP due to the cross-linking) odd number of times. The latter “parity condition” serves to discount, e.g., the cases when the linear chain just briefly intersects a ring’s surface twice, the ends being at the same side of the surface, thereby the chain not posing a significant topological obstacle for the SCNP dynamics relevant in rheology. Computing the threading of all SCNP-linear pairs in the system, with the definitions above, we get a conservative estimate of the threading topological constraints *n*_t_ being the number of linear chains threading one SCNP on average. We plot the average *n*_t_ as a function of the fraction of SCNP in Figure 6. As expected for a dense random system, n_t_ is a linear function of the composition. Interestingly, the SCNPs become slower than the linear chains at ϕ < 0.8, i.e., when the number of threadings per SCNP become significant (*n*_t_ > 1). At a high fraction of linear chains, *n*_t_ reaches values of up to 5. The large *n*_t_ > 1 strongly slows down the dynamics of the SCNPs, as they can only diffuse when all the threadings are released by reptation, confirming the experimental trends.

In ring-linear blends,^34^ the rings dominate the relaxation time (and the viscosity peaks) when the number of threadings per ring is greater than the number of linear–linear entanglements (1-ϕ)*Z*. The key message here is that replacing a linear chain by a ring means that we actually replace *Z* entanglements by *n*_t_ threadings. This replacement effectively weakens the linear entanglement network of the blend, if every ring contributes fewer constraints than linear chains,^34^ leading to a decrease of the relaxation time of the rings. To test the applicability of these arguments to SCNP-linear blends, we determined the linear–linear entanglements using the primitive path analysis (see Supporting Information and Figures S13–S14). As shown in Figure 6, the replacement of the entanglements by threadings cannot explain the dynamic crossover, as the contribution of linear–linear entanglements is predominant. As shown in the Supporting Information, another method to determine the threadings^34^ overestimates their number and also does not explain the position of the viscosity peak.

While there is a qualitative analogy with ring-linear threadings that are governed by the ring-linear constraint release (see also Figure 3),^8^ it is emphasized that SCNPs are quite different from rings. In contrast to rings, they are more heterogeneous, their size is almost insensitive to blend composition, and the composition at which their relaxation time dominates over that of linear chains is different from the one where the viscosity peaks.

## 4 Concluding Remarks

We have synthesized and characterized self-associating linear chains, known as single-chain nanoparticles, SCNP and examined the dynamic properties of their blends with linear chains. A systematic rheological study of their blends with linear entangled polymers having larger zero-shear viscosity revealed that adding such multiloop structures with a loop molar mass clearly above the Kuhn limit (here, by a factor of 6) yields a nonmonotonic concentration dependence of the blend’s viscosity. With the help of simulations, we confirm this finding and get a further insight by accessing the partial contributions (from the linear and from the SCNPs) to the dynamics. The nonmonotonic behavior originates from the interplay of the very different dependence of the partial contributions on the blend composition. The SCNPs become much slower than the linear chains as the concentration of the latter increases, which cannot be explained in terms of entanglements. This finding reflects a different topological interaction: the threading of the SCNPs by the linear chains, akin to the behavior of single-loop ring polymers added to linear matrices. This provides useful insights into entropically tailoring the viscosity of polymers by designing appropriate SCNPs.

## Supporting Information Available

Synthesis and characterization of the SCNPs; characterization of SCNPs and blends with linear chains: morphologies and molecular characteristics; threading analysis and partial intramolecular coherent scattering functions; and primitive path analysis and threading numbers (PDF)

## References

[ref1] Harth E.; Horn B. V.; Lee V. Y.; Germack D. S.; Gonzales C. P.; Miller R. D.; Hawker C. J. A Facile Approach to Architecturally Defined Nanoparticles via Intramolecular Chain Collapse. J. Am. Chem. Soc. 2002, 124 (29), 8653–8660. 10.1021/ja026208x. 12121107

[ref2] Latorre-Sánchez A.; Pomposo J. A. Recent Bioinspired Applications of Single-Chain Nanoparticles. Polym. Int. 2016, 65 (8), 855–860. 10.1002/pi.5078.

[ref3] Pomposo J. A.; Perez-Baena I.; Lo Verso F.; Moreno A. J.; Arbe A.; Colmenero J. How Far Are Single-Chain Polymer Nanoparticles in Solution from the Globular State?. ACS Macro Lett. 2014, 3 (8), 767–772. 10.1021/mz500354q. 35590711

[ref4] Pomposo J. A.; Moreno A. J.; Arbe A.; Colmenero J. Local Domain Size in Single-Chain Polymer Nanoparticles. ACS Omega 2018, 3 (8), 8648–8654. 10.1021/acsomega.8b01331. 31458995 PMC6644443

[ref5] Verde-Sesto E.; Arbe A. J.; Moreno A.; Cangialosi D.; Alegría A.; Colmenero J. A.; Pomposo J. Single-Chain Nanoparticles: Opportunities Provided by Internal and External Confinement. Mater. Horiz. 2020, 7 (9), 2292–2313. 10.1039/D0MH00846J.

[ref6] Blasco E. T.; Tuten B.; Frisch H.; Lederer A.; Barner-Kowollik C. Characterizing Single Chain Nanoparticles (SCNPs): A Critical Survey. Polym. Chem. 2017, 8 (38), 5845–5851. 10.1039/C7PY01278K.

[ref7] Arbe A.; Rubio J.; Malo de Molina P.; Maiz J.; Pomposo J. A.; Fouquet P.; Prevost S.; Juranyi F.; Khaneft M.; Colmenero J. Melts of Single-Chain Nanoparticles: A Neutron Scattering Investigation. J. Appl. Phys. 2020, 127 (4), 044305 10.1063/1.5140705.

[ref8] Parisi D.; Ahn J.; Chang T.; Vlassopoulos D.; Rubinstein M. Stress Relaxation in Symmetric Ring-Linear Polymer Blends at Low Ring Fractions. Macromolecules 2020, 53 (5), 1685–1693. 10.1021/acs.macromol.9b02536. 33518807 PMC7839933

[ref9] Maiz J.; Verde-Sesto E.; Asenjo-Sanz I.; Mangin-Thro L.; Frick B.; Pomposo J. A.; Arbe A.; Colmenero J. Disentangling Component Dynamics in an All-Polymer Nanocomposite Based on Single-Chain Nanoparticles by Quasielastic Neutron Scattering. Macromolecules 2022, 55, 2320–2332. 10.1021/acs.macromol.1c02382. 35355834 PMC8945772

[ref10] Stadler A. M.; Stingaciu L.; Radulescu A.; Holderer O.; Monkenbusch M.; Biehl R.; Richter D. Internal Nanosecond Dynamics in the Intrinsically Disordered Myelin Basic Protein. J. Am. Chem. Soc. 2014, 136 (19), 6987–6994. 10.1021/ja502343b. 24758710

[ref11] Moreno A. J.; Lo Verso F.; Arbe A.; Pomposo J. A.; Colmenero J. Concentrated Solutions of Single-Chain Nanoparticles: A Simple Model for Intrinsically Disordered Proteins under Crowding Conditions. J. Phys. Chem. Lett. 2016, 7 (5), 838–844. 10.1021/acs.jpclett.6b00144. 26894933

[ref12] Tuteja A.; Mackay M. E.; Hawker C. J.; Van Horn B.; Ho D. L. Molecular Architecture and Rheological Characterization of Novel Intramolecularly Crosslinked Polystyrene Nanoparticles. J. Polym. Sci., Part B: Polym. Phys. 2006, 44 (14), 1930–1947. 10.1002/polb.20826.

[ref13] Gury L.; Gauthier M.; Cloitre M.; Vlassopoulos D. Colloidal Jamming in Multiarm Star Polymer Melts. Macromolecules 2019, 52 (12), 4617–4623. 10.1021/acs.macromol.9b00674.

[ref14] Arbe A.; Rubio-Cervilla J.; Alegría A.; Moreno A. J.; Pomposo J. A.; Robles-Hernández B.; Malo de Molina P.; Fouquet P.; Juranyi F.; Colmenero J. Mesoscale Dynamics in Melts of Single-Chain Polymeric Nanoparticles. Macromolecules 2019, 52 (18), 6935–6942. 10.1021/acs.macromol.9b01264.

[ref15] Bae S.; Galant O.; Diesendruck C. E.; Silberstein M. N. Tailoring Single Chain Polymer Nanoparticle Thermo-Mechanical Behavior by Cross-Link Density. Soft Matter 2017, 13 (15), 2808–2816. 10.1039/C7SM00360A. 28345097

[ref16] Galant O.; Bae S.; Wang F.; Levy A.; Silberstein M. N.; Diesendruck C. E. Mechanical and Thermomechanical Characterization of Glassy Thermoplastics with Intrachain Cross-Links. Macromolecules 2017, 50 (17), 6415–6420. 10.1021/acs.macromol.7b01472.

[ref17] Galant O.; Bae S.; Silberstein M.; Diesendruck C. Highly Stretchable Polymers: Mechanical Properties Improvement by Balancing Intra- and Intermolecular Interactions. Adv. Funct. Mater. 2020, 30, 1901806 10.1002/adfm.201901806.

[ref18] Nusser K.; Schneider G. J.; Richter D. Rheology and Anomalous Flow Properties of Poly(Ethylene-Alt-Propylene)–Silica Nanocomposites. Macromolecules 2013, 46 (15), 6263–6272. 10.1021/ma3025927.

[ref19] Tuteja A.; Mackay M. E.; Hawker C. J.; Van Horn B. Effect of Ideal, Organic Nanoparticles on the Flow Properties of Linear Polymers: Non-Einstein-like Behavior. Macromolecules 2005, 38 (19), 8000–8011. 10.1021/ma050974h.

[ref20] Mackay M. E.; Dao T. T.; Tuteja A.; Ho D. L.; van Horn B.; Kim H.-C.; Hawker C. J. Nanoscale Effects Leading to Non-Einstein-like Decrease in Viscosity. Nat. Mater. 2003, 2 (11), 762–766. 10.1038/nmat999. 14566332

[ref21] Nusser K.; Schneider G. J.; Pyckhout-Hintzen W.; Richter D. Viscosity Decrease and Reinforcement in Polymer–Silsesquioxane Composites. Macromolecules 2011, 44 (19), 7820–7830. 10.1021/ma201585v.

[ref22] Chen T.; Qian H.-J.; Lu Z.-Y. Diffusion Dynamics of Nanoparticle and Its Coupling with Polymers in Polymer Nanocomposites. Chem. Phys. Lett. 2017, 687, 96–100. 10.1016/j.cplett.2017.09.010.

[ref23] Chen T.; Zhao H.-Y.; Shi R.; Lin W.-F.; Jia X.-M.; Qian H.-J.; Lu Z.-Y.; Zhang X.-X.; Li Y.-K.; Sun Z.-Y. An Unexpected N -Dependence in the Viscosity Reduction in All-Polymer Nanocomposite. Nat. Commun. 2019, 10 (1), 5552 10.1038/s41467-019-13410-z. 31804474 PMC6895191

[ref24] Segura J. L.; Martín N. O-Quinodimethanes: Efficient Intermediates in Organic Synthesis. Chem. Rev. 1999, 99 (11), 3199–3246. 10.1021/cr990011e. 11749515

[ref25] Klonos P. A.; Patelis N.; Glynos E.; Sakellariou G.; Kyritsis A. Molecular Dynamics in Polystyrene Single-Chain Nanoparticles. Macromolecules 2019, 52 (23), 9334–9340. 10.1021/acs.macromol.9b02070.

[ref26] Ferry J. D. Viscoelastic Properties of Polymers, 3rd ed.; Wiley, 1980.

[ref27] Zoller P.; Walsh D. J. Standard Pressure Volume Temperature Data for Polymers; CRC Press, 1995.

[ref28] Kremer K.; Grest G. S. Dynamics of entangled linear polymer melts: A molecular dynamics simulation. J. Chem. Phys. 1990, 92, 5057–5086. 10.1063/1.458541.

[ref29] Bačová P.; Lo Verso F.; Arbe A.; Colmenero J.; Pomposo J. A.; Moreno A. J. The Role of the Topological Constraints in the Chain Dynamics in All-Polymer Nanocomposites. Macromolecules 2017, 50 (4), 1719–1731. 10.1021/acs.macromol.6b02340.

[ref30] Smrek J.; Grosberg A. Y. Minimal Surfaces on Unconcatenated Polymer Rings in Melt. ACS Macro Lett. 2016, 5 (6), 750–754. 10.1021/acsmacrolett.6b00289. 35614671

[ref31] Smrek J.; Kremer K.; Rosa A. Threading of Unconcatenated Ring Polymers at High Concentrations: Double-Folded vs Time-Equilibrated Structures. ACS Macro Lett. 2019, 8 (2), 155–160. 10.1021/acsmacrolett.8b00828. 30800531 PMC6383510

[ref32] Brakke K. A. The surface evolver. Exp. Math. 1992, 1 (2), 141–165. 10.1080/10586458.1992.10504253.

[ref33] Paton K. An Algorithm for Finding a Fundamental Set of Cycles of a Graph. Commun. ACM 1969, 12 (9), 514–518. 10.1145/363219.363232.

[ref34] O’Connor T. C.; Ge T.; Grest G. S. Composite entanglement topology and extensional rheology of symmetric ring-linear polymer blends. J. Rheol. 2022, 66, 49–65. 10.1122/8.0000319.

