# Supplementary material for: Nonmonotonic Composition Dependence of Viscosity upon Adding Single-Chain Nanoparticles to Entangled Polymers
Source: Macromolecules. 2024 May 14;57(10):4826–32. doi: 10.1021/acs.macromol.4c00206 (PMC11191425; doi:10.1021/acs.macromol.4c00206)
Supplement: Supplementary file 1 — ma4c00206_si_001.pdf [file ma4c00206_si_001.pdf]

Supporting Information

Non-monotonic composition dependence of viscosity upon adding single-chain nanoparticles to entangled polymers

Christina Pyromali[1,2], Nikolaos Patelis[3,], Marta Cutrano[1,4], Mounika Gosika[5,6], Emmanouil Glynos[1,2], Angel J. Moreno[5,7,*], Georgios Sakellariou[3,*] Jan Smrek[8] and Dimitris Vlassopoulos[1,2,*]

[1]FORTH, Institute of Electronic Structure & Laser, Heraklion 71110, Crete, Greece

[2]Department of Materials Science and Technology, University of Crete, Heraklion 71110, Crete, Greece

[3]Department of Chemistry, National and Kapodistrian University of Athens, Panepistimiopolis Zografou, 15771, Athens, Greece

[4]Dipartimento di Ingegneria Chimica e Materiali, Università degli Studi di Cagliari, Piazza d'Armi, I-09123, Cagliari, Italy

[5]Centro de Fisica de Materiales (CSIC-UPV/EHU) and Materials Physics Center MPC, Paseo Manuel de Lardizabal 5, E-20018 San Sebastian, Spain

[6]Department of Physics, School of Advanced Sciences, Vellore Institute of Technology, Vellore, 632014 Tamil Nadu, India

[7]Donostia International Physics Center, Paseo Manuel de Lardizabal 4, E-20018 San Sebastian, Spain

[8]Faculty of Physics, University of Vienna, 1090 Vienna, Austria

Corresponding authors

* Angel Moreno: angeljose.moreno@ehu.eus

* George Sakellariou: gsakellariou@chem.uoa.gr

* Dimitris Vlassopoulos: dvlasso@iesl.forth.gr

Contents

Synthesis and characterization of the SCNPs
Characterization of SCNPs and blends with linear chains: morphologies and molecular characteristics
Threading analysis and partial intramolecular coherent scattering functions
Primitive path analysis and threading numbers

Synthesis and characterization of the SCNPs

The synthetic route was followed according to the literature.[1,2] The synthesis of the PS-co-PVBCB linear precursors was achieved through nitroxide-mediated polymerization (NMP) to ensure the random polymerization of Styrene (S) and 4-vinylbenzocyclobutene (4-VBCB) due to different reactivity ratios. The SCNPs formation was performed in dibenzyl ether solvent in high dilution under an inert atmosphere at 250 °C. The resulting SCNPs were characterized by size exclusion chromatography (SEC) in THF, ^{1}H NMR (400 MHz, $CDCl_3$), and DLS in toluene solvent.

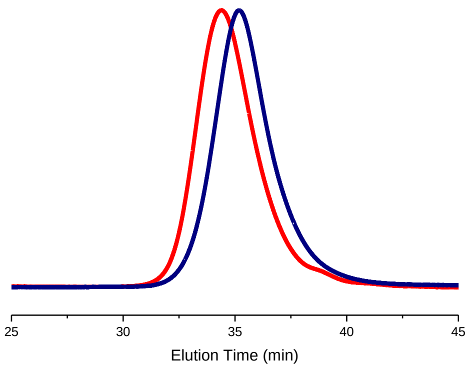

Figure S1: Size exclusion chromatography eluograms in THF, of (a) PS-*co*-PVBCB (57 kg/mol) linear polymer (blue), and the corresponding single chain nanoparticle (red).

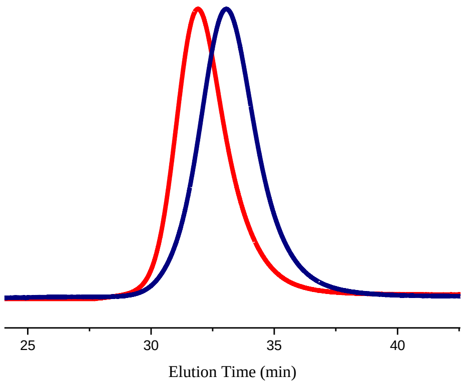

Figure S2: Size exclusion chromatography eluograms in THF, of (a) PS-*co*-PVBCB (140 kg/mol) linear polymer (blue)**,** and the corresponding single chain nanoparticle (red).

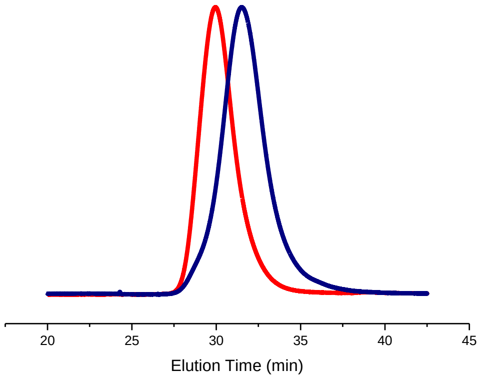

Figure S3: Size exclusion chromatography eluograms in THF, of (a) PS-*co*-P4VBCB (330 kg/mol) linear polymer (blue), and the corresponding single chain nanoparticle (red).

To check the conversion of the intra-molecular crosslinks, we obtained the ^{1}H-NMR spectra of Figure S4. The signal at 3.1-3.2 ppm which corresponds to the cyclobutene protons of the cross-linkers (S4α) disappears after the intramolecular crosslinking reaction which is strong evidence that all crosslinkers interfere during the thermal reaction (S4β).

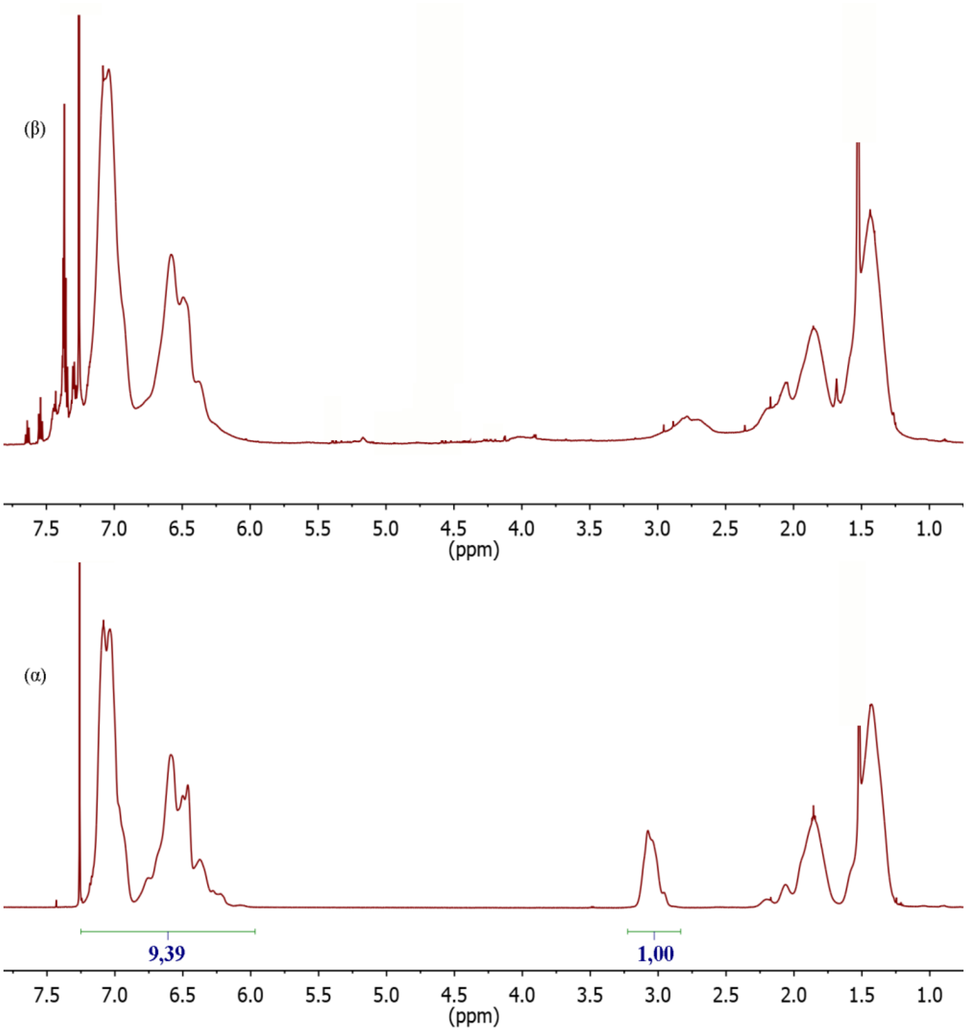

Figure S4: ^{1}H-NMR spectra of (a) PS-*co*-PVBCB linear polymer (330 kg/mol) and (α) the corresponding single chain nanoparticle. The characteristic peak of the aliphatic cyclobutene protons at 3.1-3.2 ppm (a), desapeares after the effective cross-linking reaction and the SCNP formation (β).

Characterization of SCNPs and blends with linear chains: morphologies and molecular characteristics

The average number of loops per nanoparticle (n_{loops}) and the weight-average molar mass per loop ($M_{w,\,average,loop}$) can be calculated from equations (1) and (2), respectively (the molar mass of the PS repeat unit, m_{rep}=104 gmol^{-1}):

$$n_{loops} = \frac{N^{PS} \cdot CrF}{2} \tag{1}$$

$$M_{w,\,average,loop} = N^{PS}_{average,loop} \cdot m_{rep} = \frac{2N^{PS}}{N^{CrF}} \cdot m_{rep} = \frac{2N^{PS}}{CrF \cdot N^{PS}} \cdot m_{rep} = \frac{2m_{rep}}{CrF} \tag{2}$$

Where N^{PS}, N^{CrF} and $N^{PS}_{average,loop}$ are the total number of PS and crosslinker monomers and the average degree of loop polymerization, respectively.

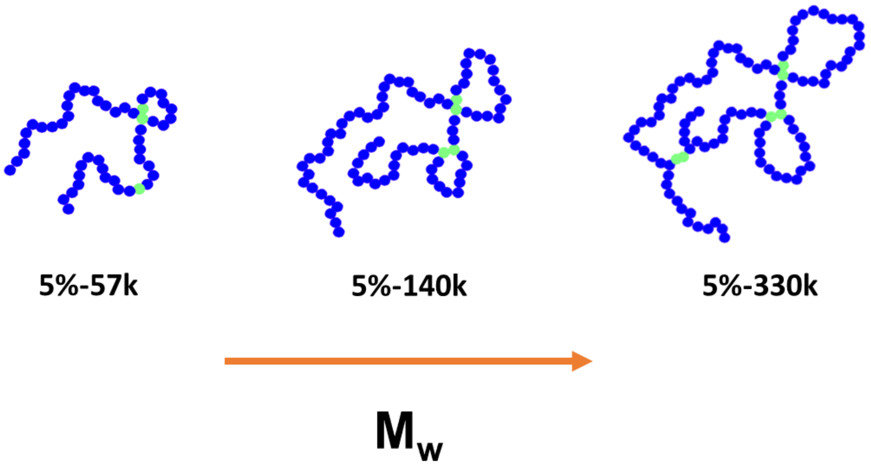

Figure S5: Schematic illustration of the synthesized SCNPs.

Thermal analysis was conducted with a DSC 250 (TA Instruments, USA). The samples (with weight between 3 and 5 mg) were encapsulated into Tzero aluminium pan-lid holders. Time constants and capacitances of the samples were determined based on the reference sensor with sapphire standard for heat capacity and indium standard for temperature and enthalpy calibration. Heating and cooling rates were fixed to 10°C/min at a temperature range from 20 to 160°C. The DSC traces revealed the glass transition temperature, T_g, at the second heating scan. The DSC traces for pure SCNPs and blends with linear polystyrene chains are provided in Figure S2 below. It should be noted that, based on the synthesis method, there should be some heterogeneity in the SCNP crosslinking density that may affect the T_g, hence the overall uncertainly in its value can be in the range 5-10%. This does not affect the present rheological results since the viscoelastic spectra are compared with respect to the same high-frequency crossover and the reported viscosities are relative (Figs. 2 and 3).

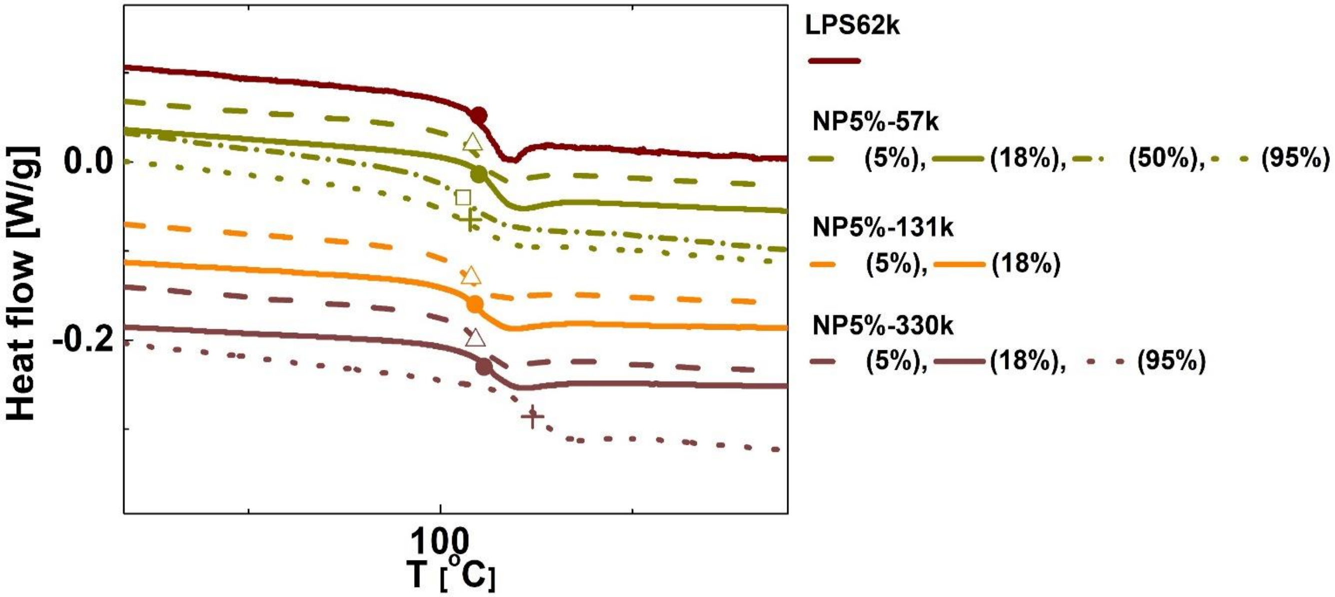

Figure S6. DSC second heating curves of pure SCNPs with rate 10 K/min. Glass transition is evaluated as the midpoint using TRIOS software. See also Table S1.

Table S1. Compositions and respective T_g values of the SCNPs-linear PS blends used.

SCNP code	ϕ (% wt)	T_g (°C)	ϕ (% wt)	T_g (°C)	ϕ (% wt)	T_g (°C)	ϕ (% wt)	T_g (°C)
5%-57k	5.1	104.2	18.1	105	50.1	103	95	103.9
5%-140k	4.7	104	18	104.5				
5%-330k	5.1	104.6	18	105.7			95	112
LPS 62k	100	105						

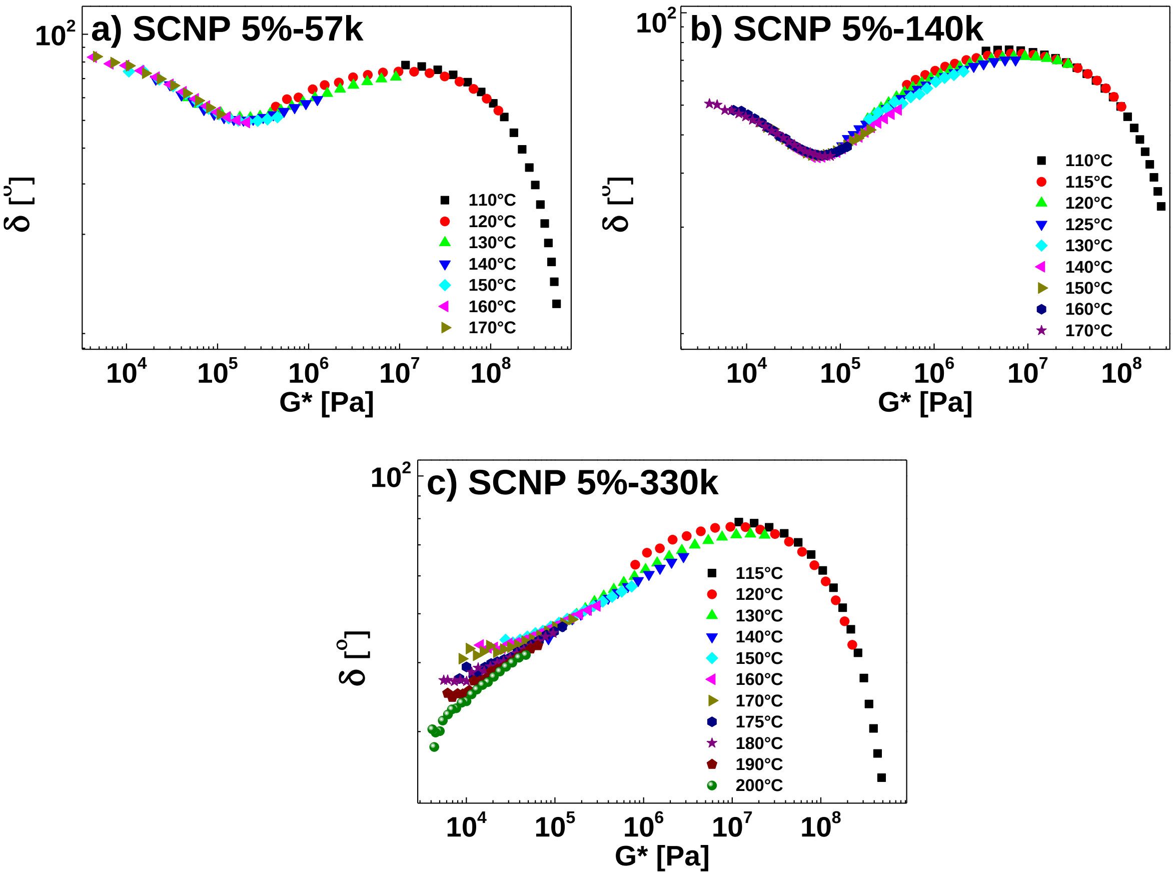

Figure S7. Temperature stability of pure SCNPs of (a) 5%-57k (b) 5%-140k and (c) 5%-330k.

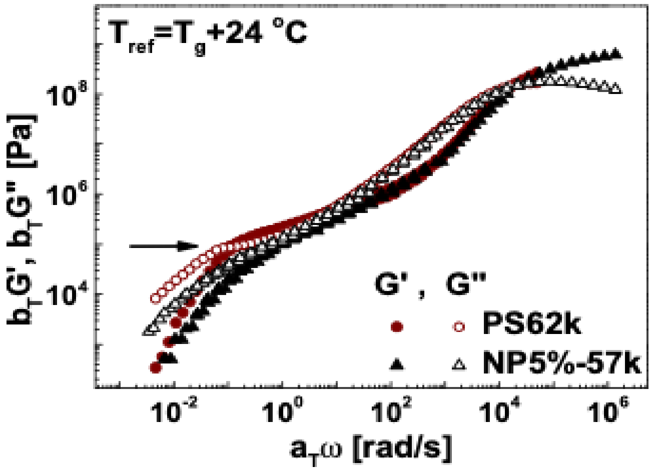

Figure S8. LVE master curves of linear polystyrene (PS62k) precursor polymer and the corresponding SCNP (75%-57k) in the melt state.

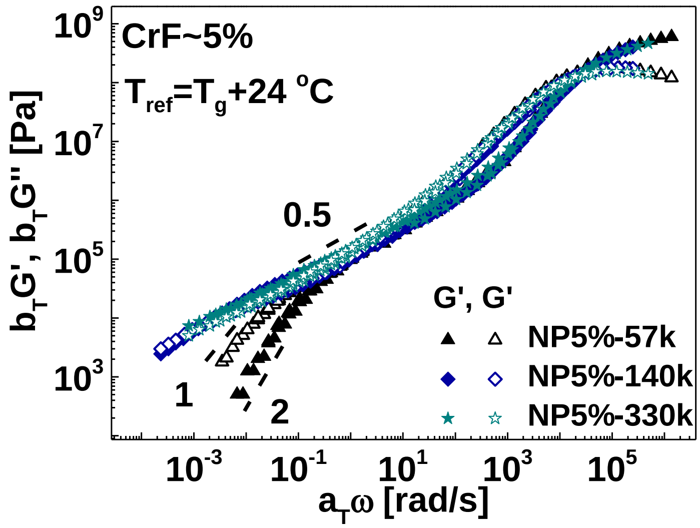

Figure S9. LVE master curves of G' (closed) and G'' (open) against angular frequency for SCNPs melts with nearly constant CrF and different parent PS-*co*-PVBCB lengths (57k, 140k & 330k) at iso-T_g condition.

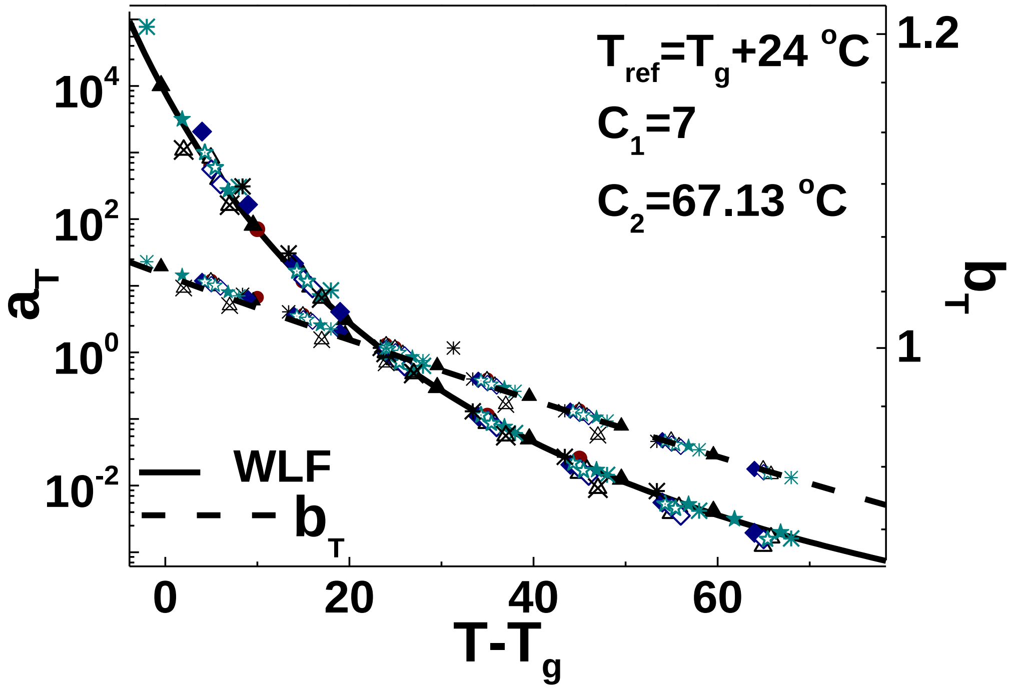

Figure S10. Horizontal (a_T) and vertical (b_T) shift factors plotted against the distance from the glass transition temperature. The lines present the WLF fit (eq. 3) and density-temperature compensation fit (eq. 4). Filled symbols correspond to PS matrix (filled red circle) and pure SCNPs (triangle 5%-57k, diamond 5%-140k, star 5%-330k) with different cross-liker fraction (CrF) and/or the length of the precursor copolymer. Unfilled symbols stand for blends of SCNPs with the linear matrix (triangle 5% SCNPs, triangle with cross18%, diamond 50%, star 95%).

Threading analysis and partial intramolecular coherent scattering functions

For visualization purposes only, the grey-colored linear chain is made "thicker", by spanning a triangular facet on every three consecutive beads.

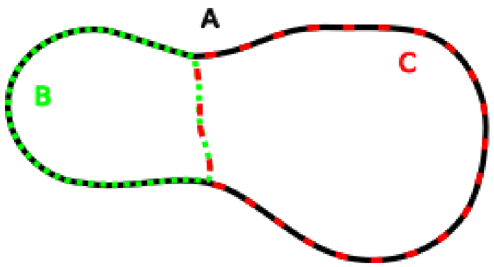

Figure S11. Schematic illustration of threadings of distinct rings from the cycle basis. Here we show the sketch of cycles. In the present example, the cycle basis is formed by two of the three color-coded cycles (**A**,**B**,**C**). Note that if the basis is formed by the cycles **A** and **B**, then whenever **B** is threaded, A is threaded too.

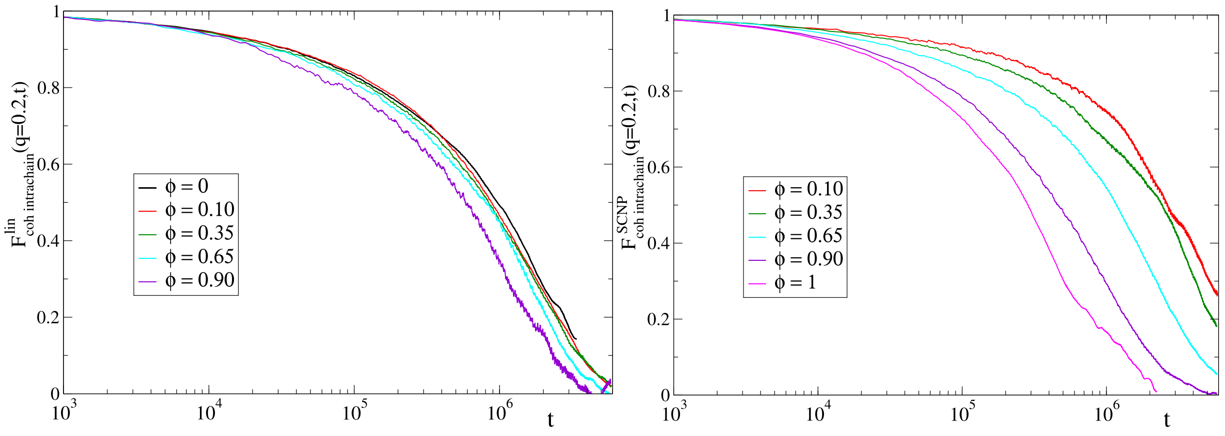

Figure S12. Partial intramolecular coherent scattering functions, averaged over all the linear chains (left) or all the SCNPs (right), and considering only pairs of beads belonging to the same molecule.

Primitive path analysis and threading numbers

We performed the PPA similarly to the one in Everaers et al.[4], where we dropped the temperature to 0.01, fixed the ends of the linear chains, removed the intrachain repulsion, but kept the interchain one. The SCNPs are left unfixed as rings in RL blends of Ref. [3], therefore they can contract to a point if not threaded. The number of entanglements Z was obtained from the linear chains only. Although Z depends on ϕ_{SCNP}, The curves (1-ϕ_{SCNP})Z(ϕ_{SCNP}) is within errorbars the same as (1-ϕ_{SCNP})Z(0). For comparison, with we computed also the number of threadings N_t as in Ref.[3] from the PPA conformations: The SCNPs that have not contracted to a point were threaded. For a threading of the SCNP by a linear chain was considered a contact of the former with the latter, but only if any of the contacting monomers of the linear chain are at least contour distance Ne from the ends. Clearly this definition overestimates the threading number as there are contacts without a real topological threadings (see a snapshot in Fig. S13).

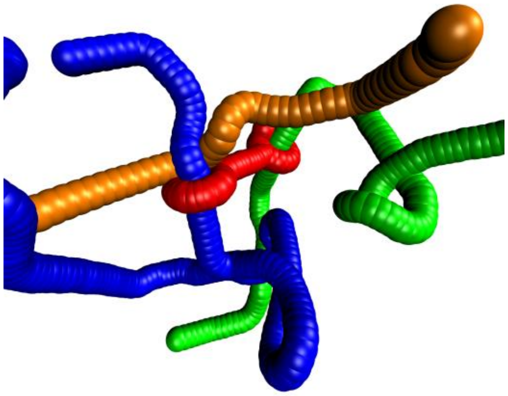

Figure S13. Snapshot from the system proving the overestimate of N_t**:** the red SCNP is threaded by blue and green linear chains. Orange linear chain is touching the SCNP too, but not threading. Other chains are not shown.

Nevertheless, in Figure S14, we compare $(1-\phi_{SCNP})Z$, n_t and N_t. As shown there the argument from Ref. [3], of the deterioration of the linear entanglement network as the fraction of SCNPs grows, is not applicable as either the number of threadings dominate or is subdominant to the linear-linear entanglements without a clear crossover. If both, the overestimate of the threading number N_t and the dependency of Z on ϕ_{SCNP} is ignored (assuming that the dilution of linear-linear entanglements is the only relevant effect), the curves cross, but somewhat in between the two critical compositions (corresponding to the viscosity peak and the relaxation time).

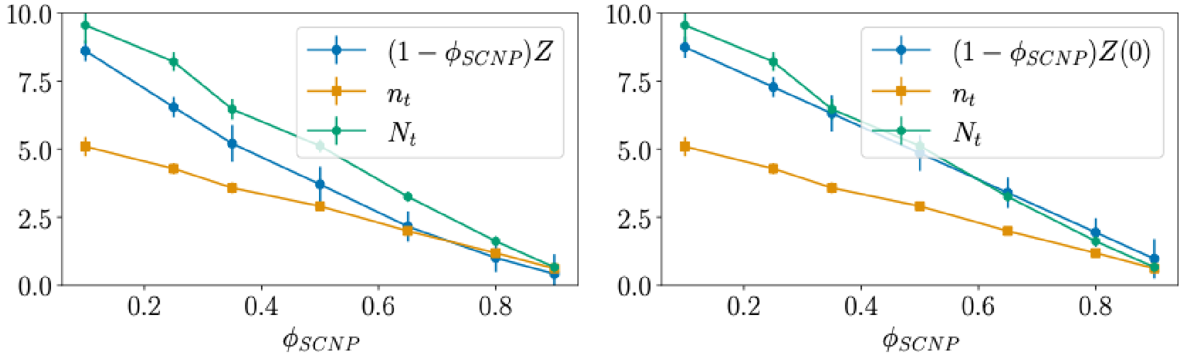

Figure S14. Comparison of threading numbers and linear-linear entanglements. Left: Z depends on ϕ_{SCNP}. Right: Only the Z at pure linear melt is considered.

References:

[1] Harth, E.; Van Horn, B.; Lee, V. Y.; Germack, D. S.; Gonzales, C. P.; Miller, R. D.; Hawker, C. J. A Facile Approach to Architecturally Defined Nanoparticles via Intramolecular Chain Collapse. *J. Am. Chem. Soc.*, **2002**, *124* (29), 8653–8660. https://doi.org/10.1021/ja026208x.

[2] Latorre-Sánchez, A.; Pomposo, J. A. Recent Bioinspired Applications of Single-Chain Nanoparticles. *Polym. Int.*, **2016**, *65* (8), 855–860. https://doi.org/10.1002/pi.5078.

[3] O'Connor, T. C.; Ge, T.; Grest, G. S. Composite entanglement topology and extensional rheology of symmetric ring-linear polymer blends, *J. Rheol*. **2022**, *66*, 49-65.

[4] Everaers, R.; Sukumaran, S. K.; Grest, G.S. ; Svaneborg, C.; Sivasubramanian, A.; Kremer, K., Rheology and microscopic topology of entangled polymeric liquids. *Science*, **2004**, 303, 823-826.
